# A new traction method – integrated multipoint traction – in endoscopic submucosal dissection for the treatment of a laterally spreading tumor

**DOI:** 10.1055/a-2519-6956

**Published:** 2025-02-11

**Authors:** Yijun Cheng, YuRong Cui, Jinxin Li, Bing Zhao, Junying Liu

**Affiliations:** 1 537323 The First Affiliated Hospital of Henan University of Traditional Chinese Medicine, Zhengzhou, China

Resection of large colorectal tumors using endoscopic submucosal dissection (ESD) remains challenging 1 . Several reports have shown that traction with a snare can be an effective aid in ESD 2 3 ; however, existing snare traction methods are constrained by a single direction and force of traction, hindering adequate exposure of the submucosal layer when applied to large laterally spreading tumors (LSTs). Our team explored a new integrated multipoint traction (IMPT) method that combines multiple endoclips and a snare to achieve all-round traction of the tumor, increasing the visibility of the submucosal layer during peeling, thereby reducing the risk of inadvertent cuts, bleeding, and perforation. This method helps to remove tumor tissue efficiently, safely, and completely.

The IMPT strategy is performed as follows. First, circumferential incision of the lesion is performed ( Fig. 1 **a** ). The endoscope is then withdrawn from the colon. Ex vivo, a snare is placed on the endoscope to co-enter the intestinal lumen to reach the lesion. Next, four endoclips are inserted sequentially through the biopsy channel, simultaneously guiding the snare and clipping it to the edges of the lesion at the 2-, 4-, 8-, and 10-oʼclock positions ( Fig. 1 **b,c** ). When the snare is tightened, the lesion is lifted in its entirety and the submucosa is adequately exposed ( Fig. 1 **d** ).

**Fig. 1:**
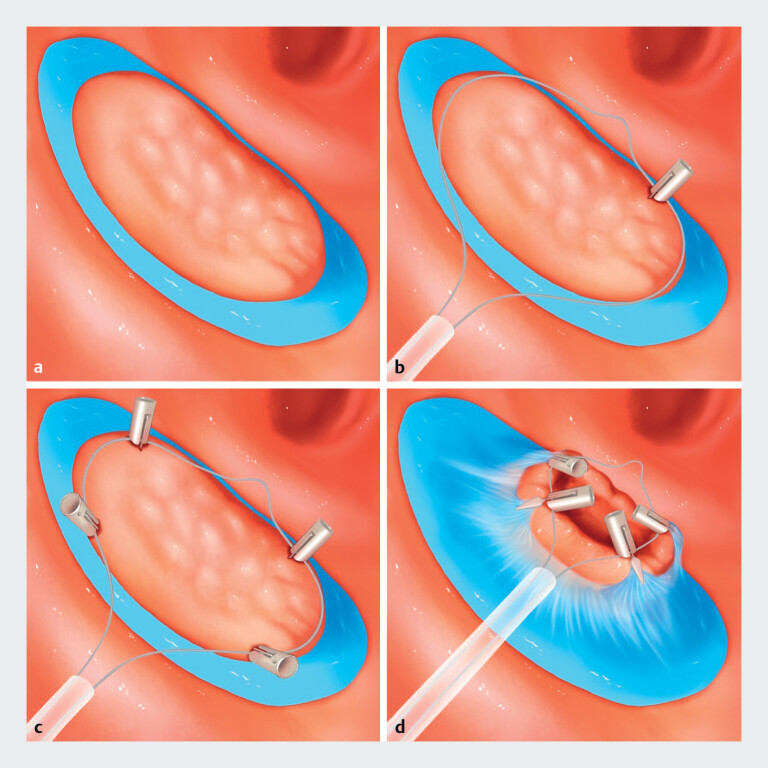
Schematic representation of the integrated multipoint traction (IMPT) technique showing: **a** circumferential incision of the lesion; **b** an endoclip being used to simultaneously guide the snare and clip it to the edge of the lesion at the 2-oʼclock position; **c** three further endoclips sequentially placed to guide and clip the snare at the 4-, 8-, and 10-oʼclock positions on the edge of the lesions; **d** adequate exposure of the submucosa when the snare is tightened, lifting the lesion in its entirety.

A 43-year-old woman was referred for ESD after being diagnosed with a 40 × 35-mm LST in the ascending colon during a screening colonoscopy ( Fig. 2 **a** ). After the target lesion had been predissected circumferentially ( Fig. 2 **b, c** ), the tumor was retracted using the IMPT strategy ( Fig. 2 **d–f** ). The lesion was completely peeled off, and the intrinsic muscular layer was protected intact without any adverse events ( Fig. 2 **g–i** ). The operative time was 25 minutes. Histopathologic examination showed a tubular adenoma with high grade intraepithelial neoplasia with negative margins ( Video 1 ).

**Fig. 2:**
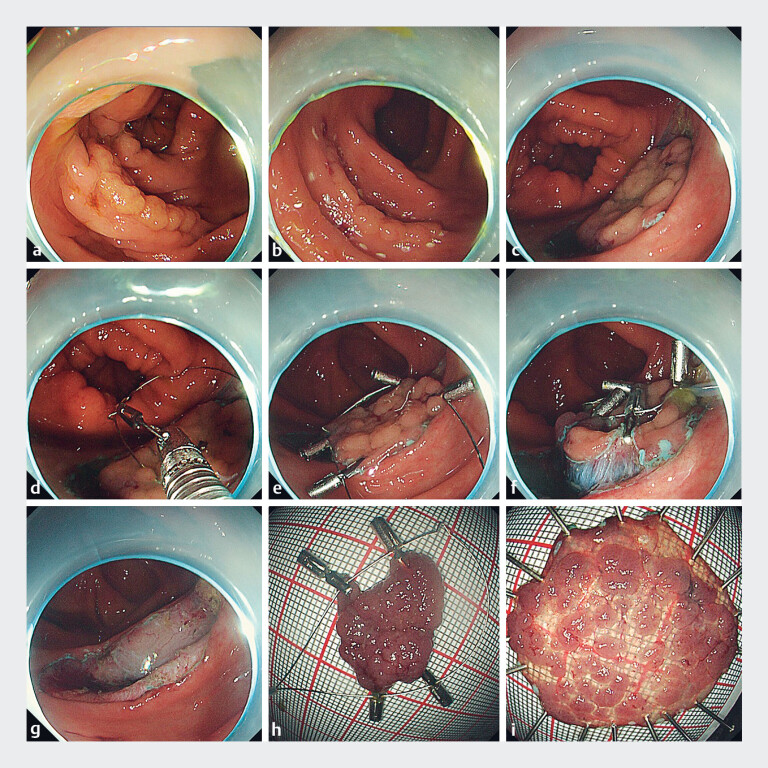
Images during colorectal endoscopic submucosal dissection for a laterally spreading tumor (LST) showing: **a** the LST, measuring approximately 40 × 35 mm, in the ascending colon; **b, c** the circumferential predissection being performed; **d–f** the application of the integrated multipoint traction (IMPT) technique; **g** the resection site, with no evidence of perforation or bleeding; **h, i** the macroscopic appearance of the resected specimen.

A new traction method, integrated multipoint traction, is used for endoscopic submucosal dissection for the treatment of a laterally spreading tumor.Video 1

In conclusion, the IMPT strategy is a new mucosal traction method, which can flexibly adjust the direction and strength of traction multiple times, maximizing the exposure of the submucosal layer and thereby reducing the difficulty and improving the efficiency of the ESD procedure.

Endoscopy_UCTN_Code_TTT_1AO_2AG_3AD

## Notes

Endoscopy E-Videos https://eref.thieme.de/e-videos
*E-Videos* is an open access online section of the journal *Endoscopy* , reporting on interesting cases and new techniques in gastroenterological endoscopy. All papers include a high-quality video and are published with a Creative Commons CC-BY license. Endoscopy E-Videos qualify for HINARI discounts and waivers and eligibility is automatically checked during the submission process. We grant 100% waivers to articles whose corresponding authors are based in Group A countries and 50% waivers to those who are based in Group B countries as classified by Research4Life (see: https://www.research4life.org/access/eligibility/ ). This section has its own submission website at https://mc.manuscriptcentral.com/e-videos .
